# Optimization of Extraction or Purification Process of Multiple Components from Natural Products: Entropy Weight Method Combined with Plackett–Burman Design and Central Composite Design

**DOI:** 10.3390/molecules26185572

**Published:** 2021-09-14

**Authors:** Yu Du, Pengcheng Huang, Weifeng Jin, Chang Li, Jiehong Yang, Haitong Wan, Yu He

**Affiliations:** 1 School of Pharmaceutical Sciences, Zhejiang Chinese Medical University, Hangzhou 310053, China; duyu970301@126.com (Y.D.); huang_pengcheng@126.com (P.H.); jin_weifeng@126.com (W.J.); 2 School of Life Sciences, Zhejiang Chinese Medical University, Hangzhou 310053, China; lichang@zcmu.edu.cn; 3 School of Basic Medicine Sciences, Zhejiang Chinese Medical University, Hangzhou 310053, China; yjhong@zcmu.edu.cn

**Keywords:** multiple-component optimization, Placket–Burman design, central composite design, macroporous resin, astragalus saponin

## Abstract

In this paper, the optimization of the extraction/purification process of multiple components was performed by the entropy weight method (EWM) combined with Plackett–Burman design (PBD) and central composite design (CCD). We took the macroporous resin purification of *Astragalus* saponins as an example to discuss the practicability of this method. Firstly, the weight of each component was given by EWM and the sum of the product between the componential content and its weight was defined as the comprehensive score, which was taken as the evaluation index. Then, the single factor method was adopted for determining the value range of each factor. PBD was applied for screening the significant factors. Important variables were further optimized by CCD to determine the optimal process parameters. After the combination of EWM, PBD and CCD, the resulting optimal purification conditions were as follows: pH value of 6.0, the extraction solvent concentration of 0.15 g/mL, and the ethanol volume fraction of 75%. Under the optimal conditions, the practical comprehensive score of recoveries of saponins was close to the predicted value (*n* = 3). Therefore, the present study provided a convenient and efficient method for extraction and purification optimization technology of multiple components from natural products.

## 1. Introduction

In recent years, optimization of extraction and purification techniques of multi components in natural products has been extensively studied and developed [1,2]. However, when a method is applied for extracting or purifying the components of natural products, many factors need to be considered. For instance, the method of macroporous adsorption resin purification has been used for the enrichment of various natural ingredients, but there are numerous conditions such as elution flow rate, sample amount and concentrations of ethanol in elution reported in the literature when separating different components [3]. For the extraction methods such as the microwave-assisted extraction method, the microwave power, heating time and amount of solvent, etc., should be taken into account [4]. Although there are multiple factors in the extraction or purification methods, only a few have significant impacts on the final results. Hence, it is necessary to establish a suitable method to screen these significant factors.

The conventional strategies for optimizing the extraction and purification method through a single-factor approach have some deficiencies. They require excessive unnecessary runs, but only give a superficial system optimum, ignoring the interaction between various factors [5]. The Plackett–Burman design (PBD) method uses a first order polynomial equation to rapidly and effectively screen the most important factors from multiple variables in a single approach [6]. Therefore, PBD can screen the conditions which possess the more significant impact among multiple conditions through a few experiments. After choosing the significant conditions, it is also necessary to determine the best parameters of the selected conditions. As a collection of mathematical and statistical techniques for modeling, response surface methodology (RSM) is usually applied to investigate the performance of complex systems and optimize the multiple process conditions [7]. Compared with the traditional single factor method or orthogonal experimental method, RSM is able to establish multiple quadratic regression equations between factors and response values to clarify the interaction among factors. It may make up for the defects by which the single factor method and orthogonal experimental method are unable to explain the interaction among factors or give the regression model between factors and response values [7,8]. Box–Behnken design (BBD) and central-composite design (CCD) are common experimental designs in RSM [9]. In the design process of CCD, there are many points that will exceed the original level. Thus, CCD better fits the response surface compared with BBD [10].

Multiple indicators affect the extraction or purification effect when facing the optimization of multiple components, so the contents of these components are usually integrated into a comprehensive score. However, the priority weights of these components are difficult to determine. The entropy weight method (EWM) is a quantitative decision analysis technique to solve the complex problems of multiple objectives. The objective weight of each indicator is assigned according to the degree of variation of various indicators. The greater the degree of variation of the index value, the more information it provides. Meanwhile, it plays greater role in the comprehensive evaluation and has greater weight [11,12,13]. Then, the comprehensive score is obtained by the sum of the product of each indicator and its weight. With this method, the content values of multiple components are integrated into a comprehensive score which considers the effect of each component.

*Astragali* radix is a popular herbal medicine which has been widely applied for over 2000 years in China. Furthermore, it is also consumed as a health natural food additive, and has great potential for the development of nutraceutical and pharmaceutical products [14]. The bulk of the commercial supply of *Astragali* radix is procured from China, of which almost 50% is used to produce *Astragali* radix decoction products and 50% is currently used for Chinese patent medicines, extracts or preparations [15]. It is vital to find an efficient and simple process to extract and purify the active components of *Astragali* radix. *Astragalus* saponins have many functional properties for the cardiovascular, cerebrovascular and immune systems. Among the saponin components with high content in *Astragali* radix, astragaloside IV possesses immune-boosting and anti-inflammatory/immune-regulatory effects, while other saponins such as isoastragaloside I and II both showed inhibitory activities against LPS-induced NO production [16]. In this paper, we took the purification of *Astragalus* saponins as an example. The recoveries of seven saponins with high content in *Astragali* radix (astragaloside I, astragaloside II, astragaloside III, astragaloside IV, astragaloside V, isoastragaloside I, isoastragaloside II) were selected to be given corresponding weights by EWM and to calculate the comprehensive score as the evaluation index. Based on the results of a single factor experiment, the applicability and scientificity of PBD combined with CCD in the extraction or purification optimization of multiple components from natural products were investigated.

## 2. Results

### 2.1. Screening of Resin Types

Ten types of resin (D101, AB-8, NKA-9, HPD-300, HPD-400, DM-130, ADS-8, S-8, X-5, H-20), which had different polarity and particle size, were investigated in static adsorption/desorption experiments in order to select the proper resin type. Table 1 presents the recovery results of *Astragalus* saponins (S_1_ to S_7_ represented astragaloside V, astragaloside IV, astragaloside III, astragaloside II, astragaloside I, isoastragaloside I, isoastragaloside II, respectively) among different macroporous resins. Compared with the other resins in a comprehensive consideration of the recovery of each saponin, AB-8 macroporous resin possessed a great advantage for the seven saponins, so it was selected to optimize the purification process of total saponins.

### 2.2. Single-Factor Experiments

**Figure 1:**
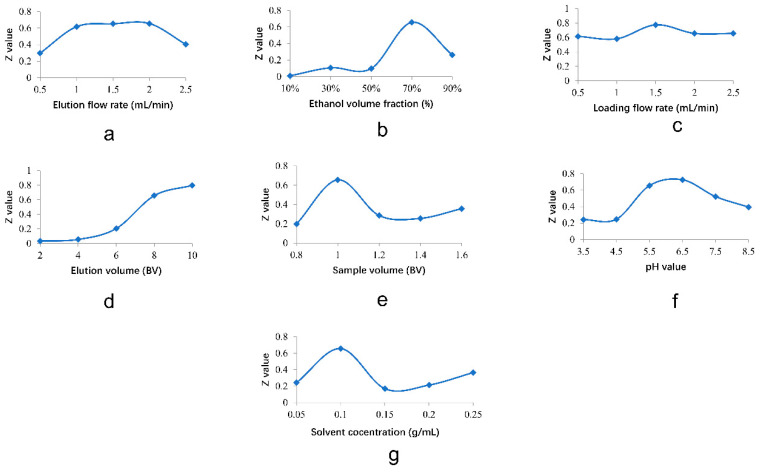
(**a**): Effect of elution volume flow rate on Z value; (**b**): effect of ethanol volume fraction on Z value; (**c**): effect of loading flow rate on Z value; (**d**): effect of elution volume on Z value; (**e**): effect of sample volume on Z value; (**f**): effect of pH value on Z value; (**g**): effect of extraction solvent on Z value.

The recovery results of the seven *Astragalus* saponins were evaluated by EWM and then the comprehensive score (Z) was calculated. The influence of each factor on Z value was shown in Figure 1.

The single factor trail showed that the comprehensive score increased firstly and then decreased with the increase of elution flow rate (Figure 1a). This result might be due to the decreased interaction between the resin and the components when the elution flow rate became too fast. The Z value was largest when the elution volume flow was 2.0 mL/min, so the elution volume flow of 2.0 mL/min was determined. The eluent selectively eluted different components when the polarity of the eluent was changed, so the comprehensive score Z was also increased firstly and then decreased (Figure 1b). The Z value for saponins reached its crest value at ethanol volume fraction of 70%, so the ethanol volume fraction of 70% was determined in the follow-up experiment. However, with the increase of sample loading flow rate, the Z value did not change obviously (Figure 1c). The result of Z value was highest at the sample flow rate of 1.5 mL/min, so the sample flow rate of 1.5 mL/min was chosen in the subsequent test. When the amount of eluent increased, the comprehensive score was elevated constantly. But this trend became slow when the volume was increased to a certain extent (Figure 1d). With the increase of the eluent volume, the impurities in the eluent had multiplied, so the elution volume of 8 BV was determined. It could be observed the Z value was highest when the ratio of sample volume to macroporous resin used was 1.0 (Figure 1e), so the ratio of 1.0 was determined. The Z value began to rise when the pH value was over 4.5 and came to a climax when the pH value was 6.5 (Figure 1f). Thus, the pH value of 6.5 was determined. With the increase of the sample solution concentration, the Z value rose and reached a maximum value of 0.656 with the concentration of 0.1 g/mL (Figure 1g). Afterwards, a downward trend was observed as the sample solution concentration increased. The sample solution concentration of 0.1 g/mL was chosen in the subsequent test.

### 2.3. Screening of Main Influencing Factors

Seven factors and four blank factors were used in this experiment. The corresponding weights of saponins were given by EWM. The weights of astragaloside I, astragaloside II, astragaloside III, astragaloside IV, astragaloside V, isoastragaloside I and isoastragaloside II were 0.145, 0.129, 0.189, 0.170, 0.085, 0.164 and 0.119, respectively. The recovery was calculated by the Z formula. PBD test results are shown in Table 2, and Table 3 is the results of variance analysis (SS represented sum of square; DF represented degree of freedom; MS represented mean square; F value represented value of F-statistic; *p* value represented value of statistical significance). According to Table 3, the obtained regression equation model was significant (*p* = 0.034), and the determination coefficient R^2^ = 93.07%, which indicate that the variability of 93.07% of the test data could be explained by this regression model. The influence of each factor on Z value was: E > D > J > G > B > H > A. E, D and J had a great influence on the comprehensive index (*p* < 0.05). Therefore, the sample solution concentration (E), the ethanol volume fraction (D) and the pH value (J) were selected as the main factors to be optimized in the purification process of saponins.

### 2.4. Central Composite Design (CCD) Results

**Figure 2:**
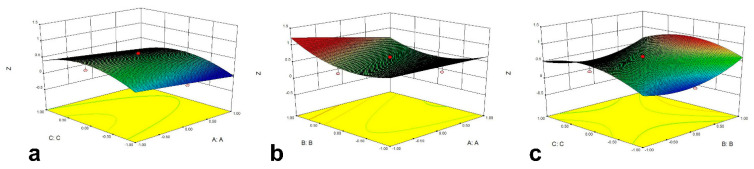
The effect of cross-interaction among pH value (X_1_), extraction solvent concentration (X_2_) and ethanol volume fraction (X_3_) on Z value. (**a**) response surface plot of effects of the interaction between pH value and ethanol volume fraction on Z value, (**b**) response surface plot of effects of the interaction between pH value and extraction solvent concentration on Z value, (**c**) response surface plot of effects of the interaction between extraction solvent concentration and ethanol volume fraction on Z value.

According to the results of PBD, three significant variables were determined. PH (X_1_), extraction solvent concentration (X_2_) and ethanol volume fraction (X_3_) were independent variables, and Z value was the dependent variable. The weight coefficients of recovery results of seven *Astragalus* saponins were given by EWM to calculate the final Z value. The low, medium and high levels of the three factors were −1, 0 and 1, respectively. The 17 experiments were performed three times each. CCD results were shown in Table 4, and the variance analysis of the response surface quadratic regression equation was listed in Table 5. The regression equation of each factor in the dynamic purification process was R = 0.53236 − 0.14629X_1_ + 0.12969X_2_ + 0.067577X_3_ − 0.11651X_1_X_2_ + 0.011643X_1_X_3_ − 0.052728X_2_X_3_ − 5.8923×10^−^^3^X_1_^2^ + 0.30519X_2_^2^ − 0.32541X_3_^2^. As Table 5 showed, the significance (*p*) of nonlinear equation model obtained by CCD was 0.0210 < 0.05, which indicated that the model was significant. The correction determination coefficient R^2^ was 0.8687, indicating that 86.87% of the variability of test data could be explained by this regression model with high reliability. The influence of each factor on Z value was X_1_ > X_2_ > X_3_, and the interaction of X_1_X_2_ in the interaction term has a certain influence on Z. Figure 2 showed the response surface diagram of X_1_, X_2_ and X_3_. Through the optimization and prediction of each factor level, the optimal purification process was obtained as the pH value of 5.95, the sample solution concentration of 0.145 g/mL, the ethanol volume fraction of 75%, and the predicted Z value was 1.0449. Considering the feasibility of this operation, the process was modified to the pH value of 6.00, the sample solution concentration of 0.15 g/mL, and the ethanol volume fraction of 75%. According to the predicted process by RSM, three batch samples were prepared and the results are shown in Table 6. From the results, the predicted value was close to the experimental value, which proved that EWM combined with PBD and CCD could be applied for the extraction or purification process optimization of multiple-components in natural products.

## 3. Materials and Methods

### 3.1. Chemical and Reagents

The standard substance of astragaloside IV (lot no. SZ20180506HQJG, ≥98%) was purchased from Shizhou Biotechnology Co., Ltd. (Nanjing, China). Acetonitrile and methanol (TEDIA, Fairfleld, OH, USA) were of HPLC grade. The analytical reagent was 95% ethanol (Huipu, Hangzhou, China). Deionized water was obtained from a Milli-Q system (Billerica, MA, USA).

*Astragali* radix (radix of *Astragalus membranaceus* (Fisch.) Bge., Lot no. 190111) was provided by Zhejiang Chinese Medical University Medical Pieces Ltd. (Hangzhou, China). Different type of Macroporous resins (D101, AB-8, NKA-9, HPD-300, HPD-400, DM-130, ADS-8, S-8, X-5, H-20) were purchased from Bon Adsorber Technology Co., Ltd. (Cangzhou, China).

Crude *Astragalus* saponins were extracted from *Astragali* radix by microwave-assisted method (RW1.5S-5E microwave dynamic extraction equipment, Orient Microwave Technology Co., Ltd., Nanjing, China). In brief, the extraction parameters were as follows, extraction time 260 s, extraction power 695 W, ethanol content 50% (*v*/*v*), the ratio of material to liquid 21.5, and the extraction times was twice. The extraction solution was evaporated to dryness and redissolved to the concentration of 0.1 g/mL with water.

### 3.2. Establishment of High-Performance Liquid Chromatography (HPLC) Fingerprint

A DGU-20A5R(C) HPLC system (Shimadzu Corporation, Japan) couple with an API 4500 Q-TRAP mass spectrometer (AB SCIEX, USA) and Agilent Infinity 1260 HPLC system (Agilent, Santa Clara, CA, USA) was used for the qualitative analysis and detection of seven *Astragalus* saponins in the solutions.

**Figure 3:**
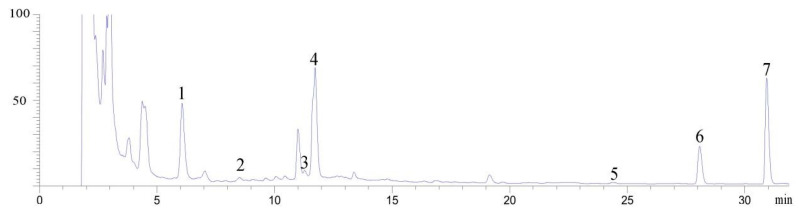
The established fingerprint of *Astragali* radix. 1-astragaloside V 2-astragaloside IV 3-astragaloside III 4-astragaloside II 5-astragaloside I 6-isoastragaloside I 7-isoastragaloside II.

The establishment of astragalosides fingerprint was referred to the previously published literature [17]. The chromatographic separation was performed at a Zorbax SB-C_18_ column (5 µm, 4.6 mm × 250 mm). The mobile phase consisted of eluent A (acetonitrile) and eluent B (water) at a flow rate of 1.0 mL/min. The gradient elution program was as follows: 35–38% A at 0~5 min, 38–50% A at 5~8 min, 50–60% A at 8~10 min, 60–70% A at 10~15 min, 70–80% A at 15~23 min, 80–95% A at 23~29 min, 95% A at 29~32 min. The column temperature was maintained at 30 °C. The injection volume was 20 μL. The wavelength was 203 nm. The established fingerprint was shown in Figure 3. Because the peak area of saponins in *Astragali* radix was proportional to their concentration, it was used to substitute for the concentration of each saponin in this experiment.

### 3.3. Entropy Weight Method

Entropy was the degree representation of the unpredictability and uncertainty of the given information in random evaluation variables. The EWM based on entropy information theory could be applied for deducing the useful information in accordance with the given data. The entropy would be low while the given information of the evaluation index possesses great significance. Thus, the given information should be considered important with a high weight coefficient [18].

Assuming there were m objects with n indexes to be evaluated in the index system, then the matric to be evaluated was as follows:

$$X={\left\{x_{ij}\right\}}_{m\times n}$$

where *x_ij_* referred to the value of the recovery of the *j*-th component in the *i*th sample (*i* = 1, 2, 3, …, *m*, *j* = 1, 2, 3, …, *n*).

The detailed formulas of EWM were as follows:

(1) Normalization of indicators:
  $$r_{ij}=(x_{ij}-\underset{i}{\min}\left\{x_{ij}\right\})/(\underset{i}{\max}\left\{x_{ij}\right\}-\underset{i}{\min}\left\{x_{ij}\right\})$$
  where *r_ij_* was the standardized value of the *j*-th component in the *i*-th sample (*i* = 1, 2, 3, …, *m*, *j* = 1, 2, 3, …, *n*).
(2) Calculation of the proportion of the *j*-th index (*p_ij_*):
  $$p_{ij}=x_{ij}/\overset{n}{\underset{i=1}{\sum }}x_{ij},\;\;i=1,\ldots ,n;j=1,\ldots ,\;m$$
(3) Calculation of the entropy value of the *j*-th index (*e_j_*):
  $$e_{j}=-k\overset{n}{\underset{i=1}{\sum }}p_{ij}\ln p_{ij},\;\;k=1/\mathrm{lnn}\;$$
(4) Calculation the information entropy redundancy of the *j*-th index (*d_j_*):
  $$d_{j}=1-e\;$$
(5) Calculation the entropy weight coefficient (*w_j_*) of the *j*-th index:
  $$w_{j}=d_{j}/\overset{m}{\underset{j=1}{\sum }}d_{j}$$

The Matlab software (MathWorks, Inc., Natick, MA, USA) was utilized to write the program of the above-mentioned entropy weight calculation process. Then the comprehensive score (*Z*) was calculated by the following formula:

$$Z=\overset{m}{\underset{i=1}{\sum }}R_{ij}w_{j}$$

*R_ij_* was the recovery of the *j*-th component in the *i*-th sample.

The peak areas of seven astragalus saponins were recorded by HPLC, and then the recovery was calculated by S = A/A_0_ × 100% (A was the peak area of each saponin in the elution solution, A_0_ was the peak area of each saponin in extraction solution).

### 3.4. Pretreatment of Macroporous Adsorption Resins

The macroporous adsorption resins were pretreated according to the reported method [19]. In order to remove the monomers and porogenic agents trapped inside the pores during the synthesis process, the resins were rinsed in 95% ethanol (*v*/*v*) for 24 h prior to the adsorption tests, and the excess ions in the resins were removed by washing with deionized water. The resins were wet-packed into a glass column (26 mm × 300 mm) according to 10 times of the amount of crude saponins.

Resins need to be regenerated after repeated use to maintain good separation. The resins were soaked in 0.4% NaOH for 6 h, washed with deionized water (until the pH of elution was 7.0), then soaked in 0.4% HCl for 6 h, and washed with deionized water (until the pH of filtrate reached 7.0). Finally, all the resins were dried at 60 °C in an electric blast drying oven to reach a constant weight.

### 3.5. Static Adsorption/Desorption Tests for Screening of Macroporous Resin Types

Different types of resin (D101, AB-8, NKA-9, HPD-300, HPD-400, DM-130, ADS-8, S-8, X-5, H-20), which had different polarity and particle size, were investigated in static adsorption/desorption experiments to select the proper resin type. We added 3 g of pretreated resins and 25 mL of 0.1 g/mL crude saponin solution to a 100 mL Erlenmeyer flask with a stopper. Then the flask was shaken in a thermostatic oscillator (SHA-A, Shanghai Double-Shun industry development Co., Ltd., Shanghai, China) at 100 r/min and 25 °C for 24 h. After reaching the adsorption equilibrium, the resins were filtered and washed with deionized water, then desorbed with 25 mL 70% ethanol solution.

### 3.6. Single Factor Experiments

#### 3.6.1. Effect of Elution Flow Rate on Saponins Purification

To investigate the effect of elution flow rate on saponins purification, the resin selected according to the static adsorption/desorption experiments was eluted by different flow rates of 0.5, 1.0, 1.5, 2.0, 2.5 mL/min, while other parameters were fixed as follows: elution volume of 6 BV, ethanol-water solution of 70% (*v*/*v*), crude saponins concentration of 0.20 g/mL, pH value of 4.5, sample flow rate of 0.5 mL/min, ratio of the crude saponins extraction solvent to the resin of 1.0.

#### 3.6.2. Effect of Ethanol Volume Fraction on Saponins Purification

The proper ethanol volume fraction was investigated through eluting samples with different ethanol-water solutions (*v*/*v*) of 10%, 30%, 50%, 70%, 90% at a flow rate of 1.5 mL/min, and the other parameters were fixed according to the aforementioned parameters.

#### 3.6.3. Effect of Sample Volume on Saponins Purification

On the basis of the constant dosage of resin, the adsorption capacity of resin for total saponins was tested at the different ratios of the crude saponins extraction solution to the resin of 0.8, 1.0, 1.2, 1.4, 1.6 respectively, and the other parameters were fixed at the aforementioned parameters.

#### 3.6.4. Effect of Elution Volume on Saponins Purification

In order to obtain the proper elution volume, the different elution volume of 2, 4, 6, 8, 10 BV were performed as other parameters were fixed at the aforementioned parameters.

#### 3.6.5. Effect of Sample Flow Rate on Saponins Purification

Adsorption capacity might be affected by sample flow rate. Based on the aforementioned parameters, the sample flow rates of 0.5, 1.0, 1.5, 2.0, 2.5 mL/min were investigated to screen the optimal sample flow rate.

#### 3.6.6. Effect of pH Value on Saponins Purification

The adsorption ability of resin is affected by pH value of extraction solution. Therefore, the effects of different pH values of 3.5, 4.5, 5.5, 6.5, 7.5, 8.5 on resin adsorption ability were evaluated under other parameters fixed at the aforementioned parameters.

#### 3.6.7. Effect of Sample Solution Concentration on Saponins Purification

The different solution concentrations of 0.05, 0.10, 0.15, 0.20, 0.25 g/mL for *Astragalus* saponins were tested to optimize the maximum adsorption capacity of resin.

### 3.7. Screening of Main Influencing Factors

The significant factors affecting the recovery of *Astragalus* saponins were screened by PBD. Each test was repeated three times and the recoveries of seven saponins were calculated. The elution flow rate (A), eluent volume (B), ethanol volume fraction (D), sample solution concentration (E), sample volume (G), sample flow rate (H), pH value (J) and four blank factors (C, F, I, K) were chosen as the experimental factors. Based on the results of a single factor experiment, Design expert 10.0.6 software (Stat-Ease Inc., Minneapolis, MN, USA) was applied to analyze the significant factors (*p* < 0.05 denoted statistical significance).

The CCD test was applied to optimize the purification process of total *Astragalus* saponins by macroporous resin. The range and center point values were based on the results of single factor experiment and PBD experiment. The data were reviewed and we exploited RSM to fit the equation.

## 4. Discussion

Aiming to enrich the components of astragalosides, 10 kinds of macroporous resin with different polarities, such as non-polarity (D101, HPD-300, H-20, X-5, ADS-8), weak polarity (CAD-40, AB-8, DM130), medium polarity (HPD-400) and strong polarity (S-8, NKA-9), were selected in this experiment. It has been reported that macroporous resin with weak polarity had a special selectivity for saponins, which was suitable for the purification of saponins or some organic substances from aqueous solution [20]. The non-polar macroporous resin had good network structure and higher specific surface area, which selectively adsorbed organic substances from aqueous solution through physical adsorption. In our research, AB-8 macroporous resin had a strong selective enrichment effect on astragalosides, it had a significant advantage in the purification of astragalosides compared with other macroporous resins.

In this experiment, the HPLC fingerprint was applied as an evaluation pattern when refining the *Astragalus* saponins. HPLC could separate the astragalosides with different polarity. The multiple-component indexes were integrated into a comprehensive index through EWD, which avoided the randomness of the subjective weight method, and hence increased the authenticity and effectiveness of the data and reflected the complex diversities of the effective components in traditional Chinese medicine.

The single factor trail showed that the Z value was increased with the increasing pH value in the acerbic condition. The Z value was decreased in a basic condition. This might be the result of the saponins acetylated to astragaloside IV in a basic situation, which caused the elution rate of other saponin components. It could be seen from the verification results that the recovery rate of astragaloside VI had exceeded 100%, which might be due to the change of pH value. Several papers have studied the influence of pH value on astragalosides stabilities. Some astragalosides (such as astragaloside I and astragaloside II) were easy to acetylate and convert into astragaloside VI [21], which leads to the recovery rate of astragaloside VI higher than 100%. The results showed that acidity had obvious influence on the stability of astragaloside I, which easily caused a lipolysis reaction and transformation into astragaloside VI and other astragaloside. This might be the reason for the low recovery rates of astragaloside I and II.

A PBD test selected appropriate high and low levels based on the results of the single factor test, which screened the more significant factors by shorter test times. However, it did not distinguish the interaction influence of various factors and the main factor effect. CCD was a two-step test design based on three factor levels, which fully considered the interaction between the factor and response, and the interaction between factors. This design overcame the defect of the orthogonal test which only obtained the optimal factor levels [22].

After three major influencing factors were selected through PBD test, the optimization process was investigated by the CCD test. The optimal process was as follows: the pH value of 6.00, the sample solution concentration of 0.15 g/mL, the ethanol volume fraction of 75%, the sample flow rate of 1.5 mL/min, 8 BV ethanol-water (75:25, *v*/*v*), the elution flow rate of 2.0 mL/min. By this method, we obtained the purified astragalosides selectively.

## 5. Conclusions

In this paper, the multi-objective purification process was optimized by a HPLC fingerprint combined with EWM, PBD and CCD. We took the purification process of astragalosides as an example. EWD integrated the multiple-component indexes into a comprehensive index, avoiding the randomness of the subjective weight method and increasing the authenticity and effectiveness of the data. The PBD test selected appropriate high and low levels based on the results of a single factor test, which screened the more significant factors by shorter test times. The optimization process was investigated by a CCD test. The results revealed that the predicted value was close to the experimental value. It proved that EWM combined with PBD and CCD was accurate to apply to the extraction or purification process optimization of multiple components in a natural product. Hence, this method provided a new reference for the extraction and separation process optimization of natural components or the effective parts of natural products with complex components.

## Figures and Tables

**Table 1:** The recoveries of *Astragalus* saponins among different macroporous resins.

	Recovery %						
	S_1_	S_2_	S_3_	S_4_	S_5_	S_6_	S_7_
NKA-9	50%	27%	8%	4%	62%	9%	1%
ADS-8	53%	17%	8%	4%	80%	3%	3%
H-20	58%	70%	61%	25%	81%	83%	86%
DM-130	53%	42%	13%	8%	55%	1%	2%
S-8	47%	34%	9%	6%	68%	0%	1%
X-5	70%	86%	42%	56%	66%	97%	96%
HPD-300	57%	78%	71%	30%	71%	83%	76%
HPD-400	48%	30%	8%	4%	56%	1%	2%
D101	84%	93%	18%	85%	64%	77%	89%
AB-8	82%	92%	99%	90%	50%	83%	95%

**Table 2:** Each factor level and Z value in Plackett–Burman design (PBD) test.

No.	A Elution Flow Rate mL/min	B Eluent Volume BV	C	D Ethanol Volume Fraction %	E Extraction Solvent Concentration g/mL	F	G Sample Volume	H Sample Flow Rate mL/min	I	J PH Value	K	*Z*
1	1.5	8	-	50%	1	-	0.8	1.5	-	6.5	-	0.1033
2	2	10	-	70%	1	-	0.8	1	-	6.5	-	0.7906
3	1.5	8	-	70%	0.5	-	1	1	-	6.5	-	0.0703
4	2	8	-	70%	1	-	0.8	1	-	5.5	-	0.7632
5	2	10	-	50%	0.5	-	1	1	-	6.5	-	0.3425
6	2	10	-	50%	0.5	-	0.8	1.5	-	5.5	-	0.0851
7	1.5	10	-	50%	1	-	1	1	-	5.5	-	0.1267
8	1.5	10	-	70%	1	-	1	1.5	-	5.5	-	0.2229
9	1.5	8	-	50%	0.5	-	0.8	1	-	5.5	-	0.7976
10	2	8	-	50%	1	-	1	1.5	-	6.5	-	0.2116
11	1.5	10	-	70%	0.5	-	0.8	1.5	-	6.5	-	0.3805
12	2	8	-	70%	0.5	-	1	1.5	-	5.5	-	0.4791

**Table 3:** Variance analysis of PBD test.

Factor	SS	DF	MS	F Value	*p* Value
Model	0.814189	7	0.116313	7.68	0.034
A	0.004005	1	0.004005	0.26	0.634
B	0.050962	1	0.050962	3.36	0.141
D	0.221486	1	0.221486	14.62	0.019
E	0.24471	1	0.24471	16.15	0.016
G	0.06952	1	0.06952	4.59	0.099
H	0.010557	1	0.010557	0.7	0.451
J	0.21295	1	0.21295	14.05	0.02
Residual	0.060609	4	0.015152		
Cor total	0.874798	11			

**Table 4:** Each factor level and Z value in central composite design (CCD) test.

	X_1_	X_2_	X_3_	Z
1	0	−1	0	0.5452
2	1	−1	−1	0.2407
3	0	0	0	0.6756
4	−1	0	0	0.4996
5	1	0	0	0.3569
6	1	−1	1	0.4966
7	0	0	1	0.1344
8	−1	1	−1	0.9266
9	1	1	−1	0.3113
10	0	0	0	0.6674
11	0	1	0	0.9335
12	−1	1	1	0.9251
13	0	0	0	0.6466
14	1	1	1	0.4142
15	−1	−1	1	0.5993
16	−1	−1	−1	0.3321
17	0	0	−1	0.0831

**Table 5:** The variance analysis of response surface quadratic regression equation.

Factor	SS	DF	MF	F Value	*p* Value
Model	0.96	9	0.11	5.15	0.021
A	0.21	1	0.21	10.33	0.0148
B	0.17	1	0.17	8.12	0.0247
C	0.046	1	0.046	2.2	0.1812
AB	0.11	1	0.11	5.24	0.0558
AC	1.085 × 10^−3^	1	1.085 × 10^−3^	0.052	0.8255
BC	0.022	1	0.022	1.07	0.3346
A^2^	9.302 × 10^−5^	1	9.302 × 10^−5^	4.491 × 10^−3^	0.9484
B^2^	0.25	1	0.25	12.05	0.0104
C^2^	0.28	1	0.28	13.7	0.0076
Residual	0.14	7	0.021		
Lack of fit	0.14	5	0.029	129.28	0.0077
Pure Error	4.472 × 10^−4^	2	2.236 × 10^−4^		
Cor total	1.1	16			

**Table 6:** Verification of response surface analysis method.

	S_1_	S_2_	S_3_	S_4_	S_5_	S_6_	S_7_	Z	RSD %
CCD	98.17%	95.47%	116.91%	95.22%	89.79%	96.37%	99.71%	1.0017	0.15%
	98.38%	95.63%	117.76%	95.59%	89.13%	97.42%	98.40%	1.0041	
	98.21%	96.70%	118.30%	95.92%	88.39%	96.79%	99.21%	1.0047	
Weight	0.123	0.146	0.219	0.124	0.162	0.104	0.122		

## Data Availability

All data is contained within the article.

## Sample Availability

Samples of the compounds are available from the authors.
